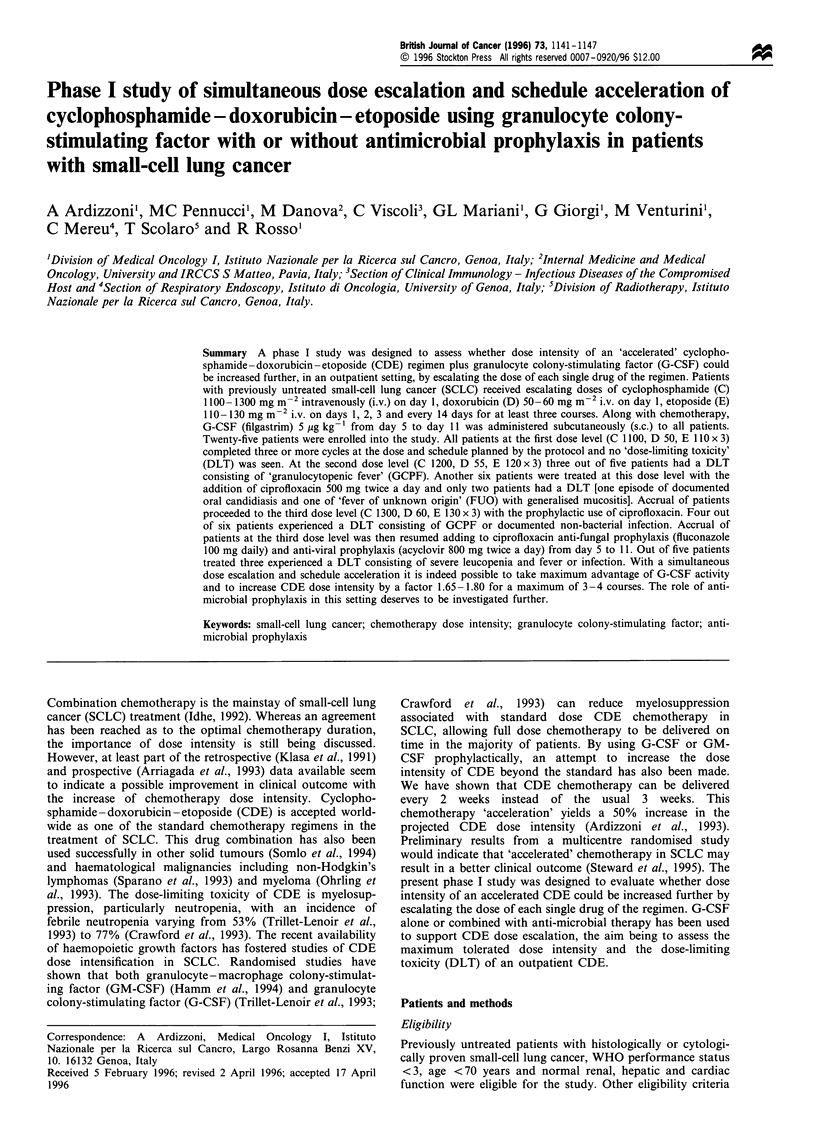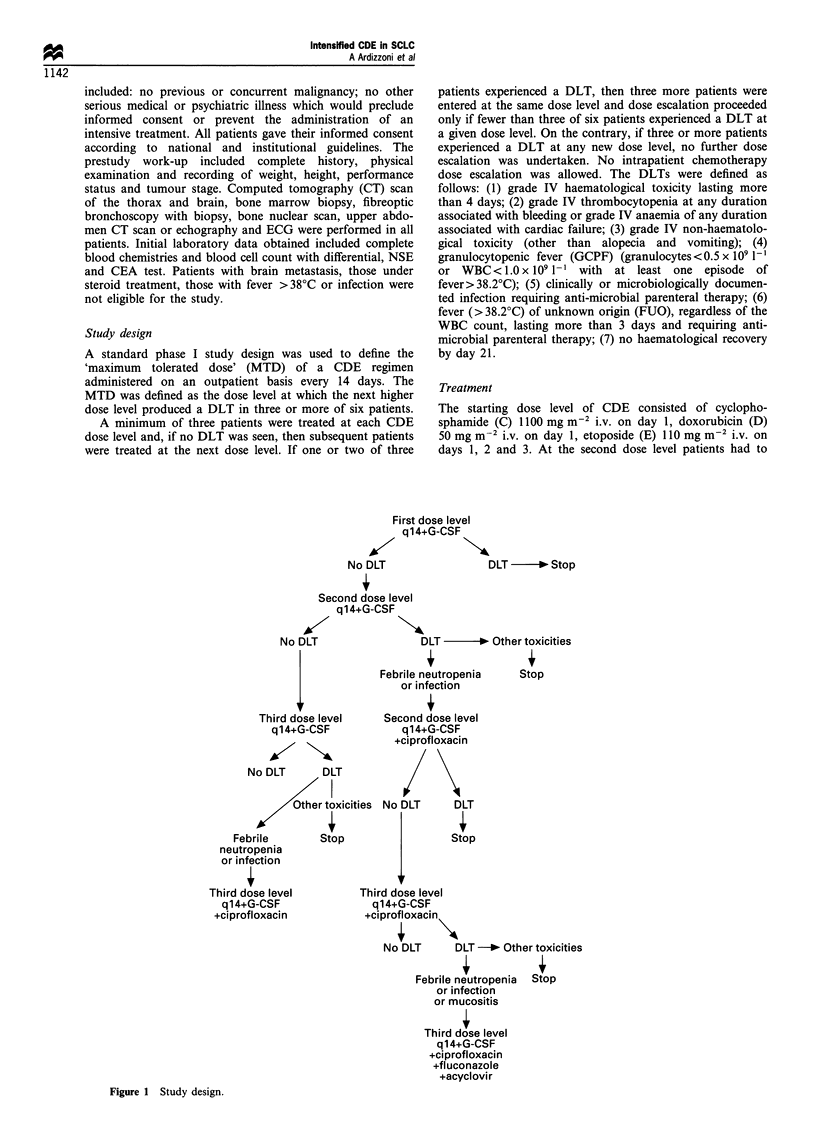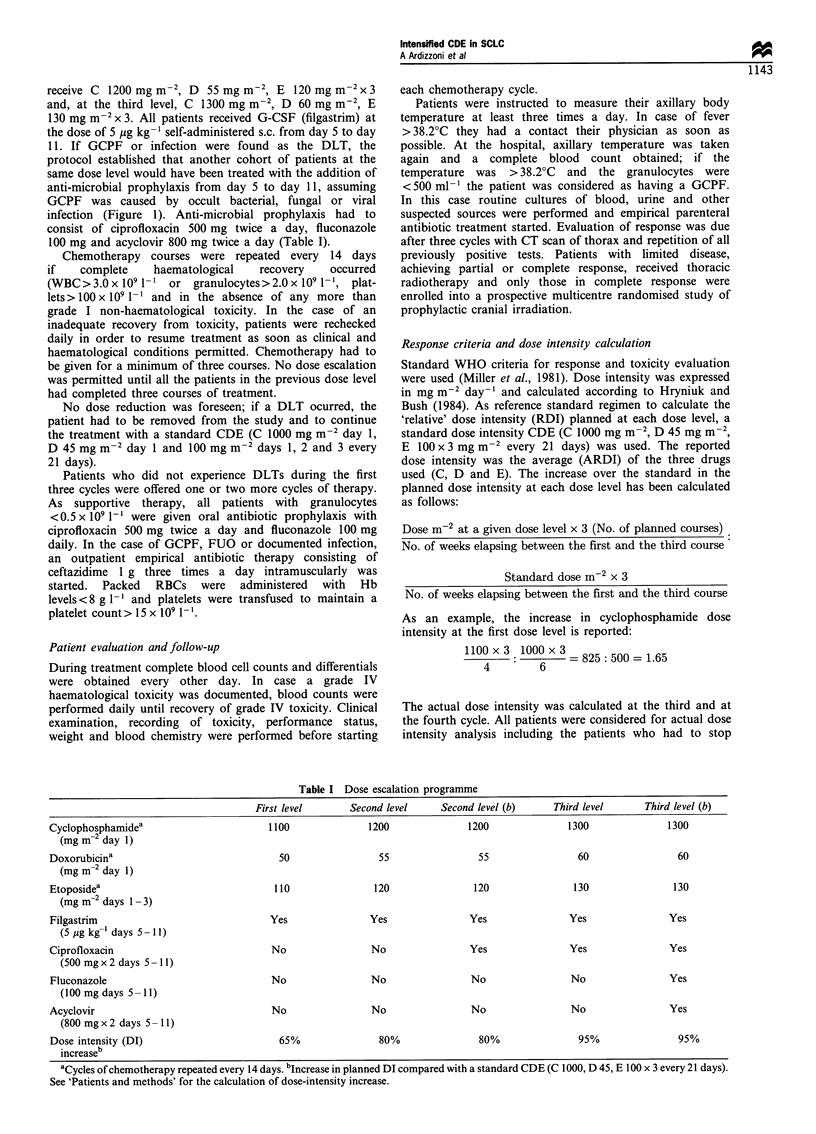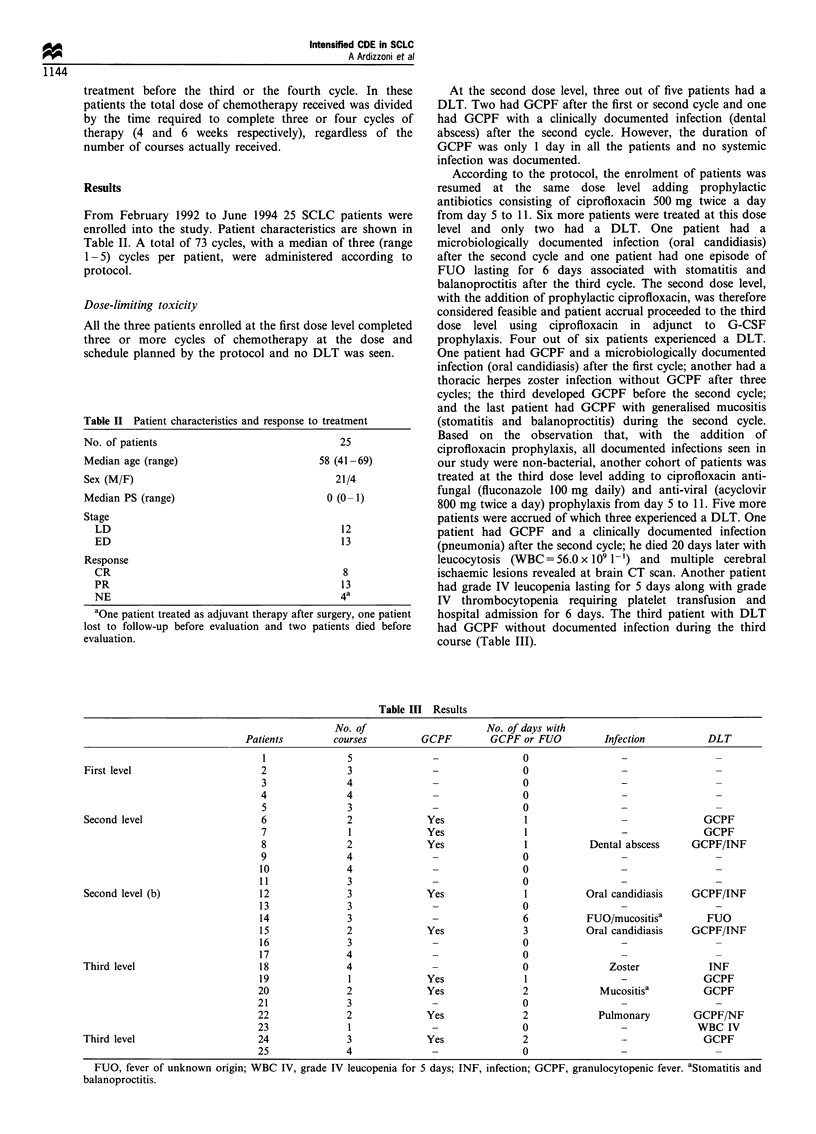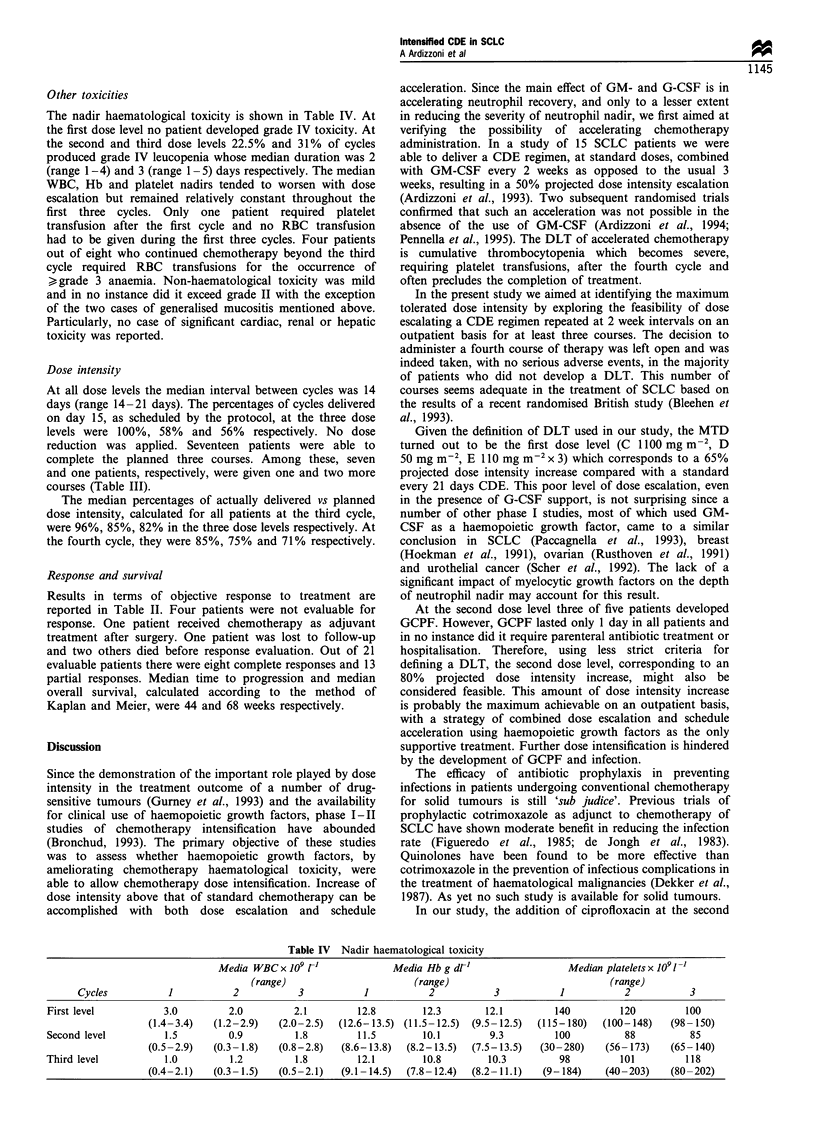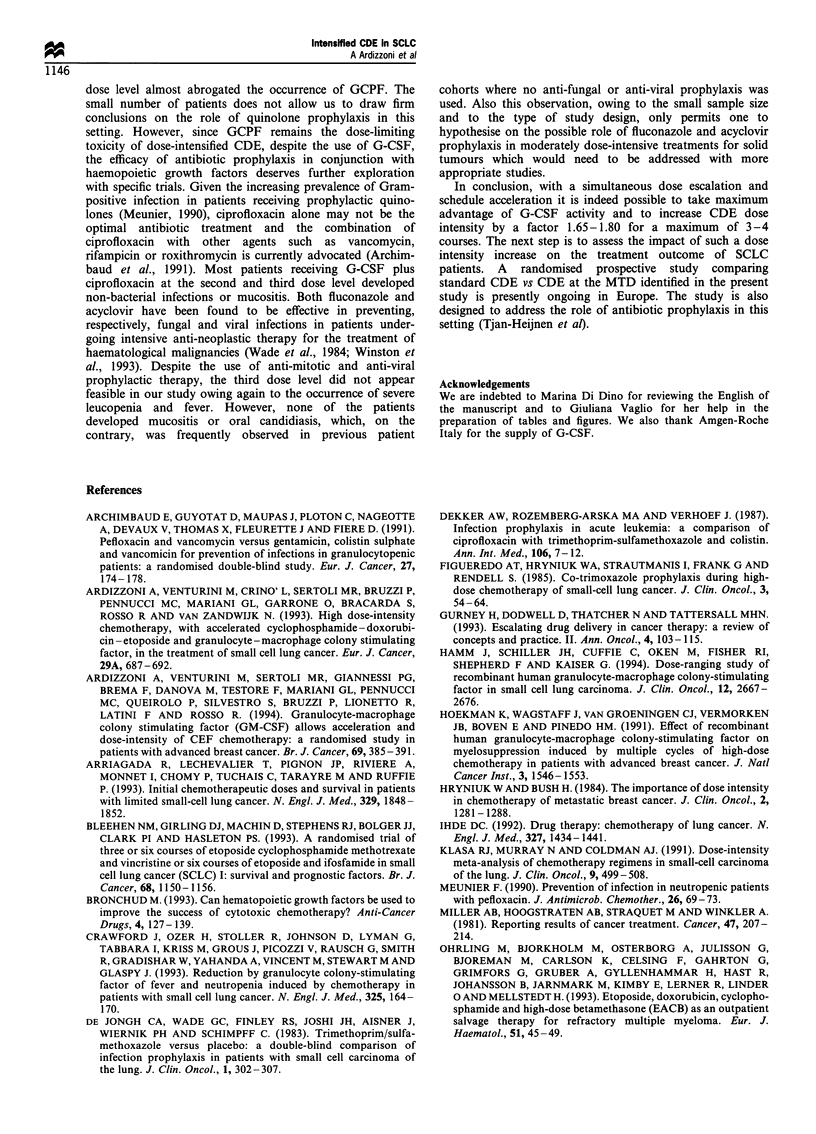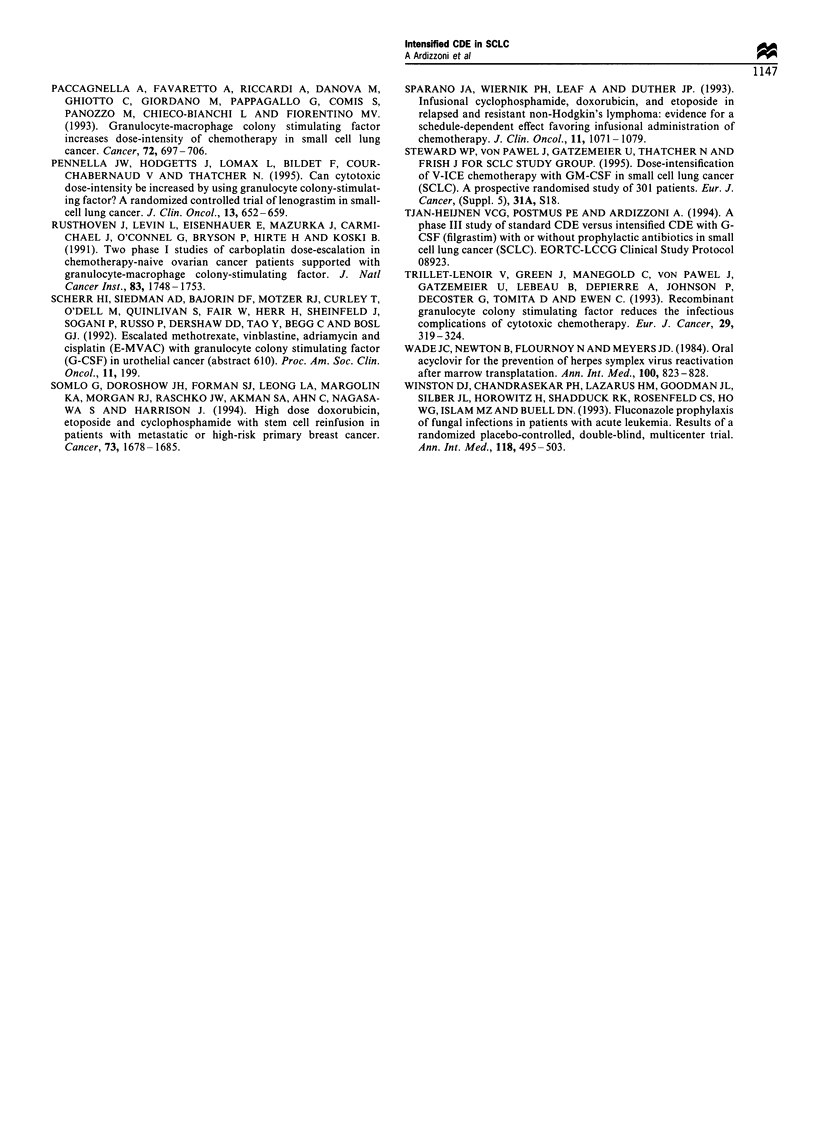# Phase I study of simultaneous dose escalation and schedule acceleration of cyclophosphamide-doxorubicin-etoposide using granulocyte colony-stimulating factor with or without antimicrobial prophylaxis in patients with small-cell lung cancer.

**DOI:** 10.1038/bjc.1996.504

**Published:** 1996-10

**Authors:** A. Ardizzoni, M. C. Pennucci, M. Danova, C. Viscoli, G. L. Mariani, G. Giorgi, M. Venturini, C. Mereu, T. Scolaro, R. Rosso

**Affiliations:** Division of Medical Oncology I, Istituto Nazionale per la Ricerca sul Cancro, Genoa, Italy.

## Abstract

A phase I study was designed to assess whether dose intensity of an 'accelerated' cyclophosphamide-doxorubicin-etoposide (CDE) regimen plus granulocyte colony-stimulating factor (G-CSF) could be increased further, in an outpatient setting, by escalating the dose of each single drug of the regimen. Patients with previously untreated small-cell lung cancer (SCLC) received escalating doses of cyclophosphamide (C) 1100-1300 mg m-2 intravenously (i.v.) on day 1, doxorubicin (D) 50-60 mg m-2 i.v. on day 1, etoposide (E) 110-130 mg m-2 i.v. on days 1, 2, 3 and every 14 days for at least three courses. Along with chemotherapy, G-CSF (filgastrim) 5 micrograms kg-1 from day 5 to day 11 was administered subcutaneously (s.c.) to all patients. Twenty-five patients were enrolled into the study. All patients at the first dose level (C 1100, D 50, E 110 x 3) completed three or more cycles at the dose and schedule planned by the protocol and no 'dose-limiting toxicity' (DLT) was seen. At the second dose level (C 1200, D 55, E 120 x 3) three out of five patients had a DLT consisting of 'granulocytopenic fever' (GCPF). Another six patients were treated at this dose level with the addition of ciprofloxacin 500 mg twice a day and only two patients had a DLT [one episode of documented oral candidiasis and one of 'fever of unknown origin' (FUO) with generalised mucositis]. Accrual of patients proceeded to the third dose level (C 1300, D 60, E 130 x 3) with the prophylactic use of ciprofloxacin. Four out of six patients experienced a DLT consisting of GCPF or documented non-bacterial infection. Accrual of patients at the third dose level was then resumed adding to ciprofloxacin anti-fungal prophylaxis (fluconazole 100 mg daily) and anti-viral prophylaxis (acyclovir 800 mg twice a day) from day 5 to 11. Out of five patients treated three experienced a DLT consisting of severe leucopenia and fever or infection. With a simultaneous dose escalation and schedule acceleration it is indeed possible to take maximum advantage of G-CSF activity and to increase CDE dose intensity by a factor 1.65-1.80 for a maximum of 3-4 courses. The role of antimicrobial prophylaxis in this setting deserves to be investigated further.


British Journal of Cancer (1996) 73, 1141-1147
© 1996 Stockton Press All rights reserved 0007-0920/96 $12.00

### Phase I study of simultaneous dose escalation and schedule acceleration of cyclophosphamide–doxorubicin–etoposide using granulocyte colony-stimulating factor with or without antimicrobial prophylaxis in patients with small-cell lung cancer

A Ardizzoni[1], MC Pennucci[1], M Danova[2], C Viscoli[3], GL Mariani[1], G Giorgi[1], M Venturini[1], C Mereu[4], T Scolaro[5] and R Rosso[1]

*[1]Division of Medical Oncology I, Istituto Nazionale per la Ricerca sul Cancro, Genoa, Italy; [2]Internal Medicine and Medical Oncology, University and IRCCS S Matteo, Pavia, Italy; [3]Section of Clinical Immunology – Infectious Diseases of the Compromised Host and [4]Section of Respiratory Endoscopy, Istituto di Oncologia, University of Genoa, Italy; [5]Division of Radiotherapy, Istituto Nazionale per la Ricerca sul Cancro, Genoa, Italy.*

**Summary** A phase I study was designed to assess whether dose intensity of an 'accelerated' cyclophosphamide–doxorubicin–etoposide (CDE) regimen plus granulocyte colony-stimulating factor (G-CSF) could be increased further, in an outpatient setting, by escalating the dose of each single drug of the regimen. Patients with previously untreated small-cell lung cancer (SCLC) received escalating doses of cyclophosphamide (C) 1100–1300 mg $m^{-2}$ intravenously (i.v.) on day 1, doxorubicin (D) 50–60 mg $m^{-2}$ i.v. on day 1, etoposide (E) 110–130 mg $m^{-2}$ i.v. on days 1, 2, 3 and every 14 days for at least three courses. Along with chemotherapy, G-CSF (filgastrim) 5 $\mu g$ $kg^{-1}$ from day 5 to day 11 was administered subcutaneously (s.c.) to all patients. Twenty-five patients were enrolled into the study. All patients at the first dose level (C 1100, D 50, E 110 × 3) completed three or more cycles at the dose and schedule planned by the protocol and no 'dose-limiting toxicity' (DLT) was seen. At the second dose level (C 1200, D 55, E 120 × 3) three out of five patients had a DLT consisting of 'granulocytopenic fever' (GCPF). Another six patients were treated at this dose level with the addition of ciprofloxacin 500 mg twice a day and only two patients had a DLT [one episode of documented oral candidiasis and one of 'fever of unknown origin' (FUO) with generalised mucositis]. Accrual of patients proceeded to the third dose level (C 1300, D 60, E 130 × 3) with the prophylactic use of ciprofloxacin. Four out of six patients experienced a DLT consisting of GCPF or documented non-bacterial infection. Accrual of patients at the third dose level was then resumed adding to ciprofloxacin anti-fungal prophylaxis (fluconazole 100 mg daily) and anti-viral prophylaxis (acyclovir 800 mg twice a day) from day 5 to 11. Out of five patients treated three experienced a DLT consisting of severe leucopenia and fever or infection. With a simultaneous dose escalation and schedule acceleration it is indeed possible to take maximum advantage of G-CSF activity and to increase CDE dose intensity by a factor 1.65–1.80 for a maximum of 3–4 courses. The role of antimicrobial prophylaxis in this setting deserves to be investigated further.

**Keywords:** small-cell lung cancer; chemotherapy dose intensity; granulocyte colony-stimulating factor; antimicrobial prophylaxis

Combination chemotherapy is the mainstay of small-cell lung cancer (SCLC) treatment (Idhe, 1992). Whereas an agreement has been reached as to the optimal chemotherapy duration, the importance of dose intensity is still being discussed. However, at least part of the retrospective (Klasa *et al.*, 1991) and prospective (Arriagada *et al.*, 1993) data available seem to indicate a possible improvement in clinical outcome with the increase of chemotherapy dose intensity. Cyclophosphamide–doxorubicin–etoposide (CDE) is accepted worldwide as one of the standard chemotherapy regimens in the treatment of SCLC. This drug combination has also been used successfully in other solid tumours (Somlo *et al.*, 1994) and haematological malignancies including non-Hodgkin's lymphomas (Sparano *et al.*, 1993) and myeloma (Ohrling *et al.*, 1993). The dose-limiting toxicity of CDE is myelosuppression, particularly neutropenia, with an incidence of febrile neutropenia varying from 53% (Trillet-Lenoir *et al.*, 1993) to 77% (Crawford *et al.*, 1993). The recent availability of haemopoietic growth factors has fostered studies of CDE dose intensification in SCLC. Randomised studies have shown that both granulocyte–macrophage colony-stimulating factor (GM-CSF) (Hamm *et al.*, 1994) and granulocyte colony-stimulating factor (G-CSF) (Trillet-Lenoir *et al.*, 1993; Crawford *et al.*, 1993) can reduce myelosuppression associated with standard dose CDE chemotherapy in SCLC, allowing full dose chemotherapy to be delivered on time in the majority of patients. By using G-CSF or GM-CSF prophylactically, an attempt to increase the dose intensity of CDE beyond the standard has also been made. We have shown that CDE chemotherapy can be delivered every 2 weeks instead of the usual 3 weeks. This chemotherapy 'acceleration' yields a 50% increase in the projected CDE dose intensity (Ardizzoni *et al.*, 1993). Preliminary results from a multicentre randomised study would indicate that 'accelerated' chemotherapy in SCLC may result in a better clinical outcome (Steward *et al.*, 1995). The present phase I study was designed to evaluate whether dose intensity of an accelerated CDE could be increased further by escalating the dose of each single drug of the regimen. G-CSF alone or combined with anti-microbial therapy has been used to support CDE dose escalation, the aim being to assess the maximum tolerated dose intensity and the dose-limiting toxicity (DLT) of an outpatient CDE.

Correspondence: A Ardizzoni, Medical Oncology I, Istituto Nazionale per la Ricerca sul Cancro, Largo Rosanna Benzi XV, 10. 16132 Genoa, Italy
Received 5 February 1996; revised 2 April 1996; accepted 17 April 1996

### Patients and methods

### *Eligibility*

Previously untreated patients with histologically or cytologically proven small-cell lung cancer, WHO performance status <3, age <70 years and normal renal, hepatic and cardiac function were eligible for the study. Other eligibility criteria included: no previous or concurrent malignancy; no other serious medical or psychiatric illness which would preclude informed consent or prevent the administration of an intensive treatment. All patients gave their informed consent according to national and institutional guidelines. The prestudy work-up included complete history, physical examination and recording of weight, height, performance status and tumour stage. Computed tomography (CT) scan of the thorax and brain, bone marrow biopsy, fibreoptic bronchoscopy with biopsy, bone nuclear scan, upper abdomen CT scan or echography and ECG were performed in all patients. Initial laboratory data obtained included complete blood chemistries and blood cell count with differential, NSE and CEA test. Patients with brain metastasis, those under steroid treatment, those with fever >38°C or infection were not eligible for the study.

*Study design*

A standard phase I study design was used to define the 'maximum tolerated dose' (MTD) of a CDE regimen administered on an outpatient basis every 14 days. The MTD was defined as the dose level at which the next higher dose level produced a DLT in three or more of six patients.

A minimum of three patients were treated at each CDE dose level and, if no DLT was seen, then subsequent patients were treated at the next dose level. If one or two of three patients experienced a DLT, then three more patients were entered at the same dose level and dose escalation proceeded only if fewer than three of six patients experienced a DLT at a given dose level. On the contrary, if three or more patients experienced a DLT at any new dose level, no further dose escalation was undertaken. No intrapatient chemotherapy dose escalation was allowed. The DLTs were defined as follows: (1) grade IV haematological toxicity lasting more than 4 days; (2) grade IV thrombocytopenia at any duration associated with bleeding or grade IV anaemia of any duration associated with cardiac failure; (3) grade IV non-haematological toxicity (other than alopecia and vomiting); (4) granulocytopenic fever (GCPF) (granulocytes $<0.5\times10^9\ l^{-1}$ or WBC $<1.0\times10^9\ l^{-1}$ with at least one episode of fever $>38.2$°C); (5) clinically or microbiologically documented infection requiring anti-microbial parenteral therapy; (6) fever ($>38.2$°C) of unknown origin (FUO), regardless of the WBC count, lasting more than 3 days and requiring anti-microbial parenteral therapy; (7) no haematological recovery by day 21.

*Treatment*

The starting dose level of CDE consisted of cyclophosphamide (C) 1100 mg $m^{-2}$ i.v. on day 1, doxorubicin (D) 50 mg $m^{-2}$ i.v. on day 1, etoposide (E) 110 mg $m^{-2}$ i.v. on days 1, 2 and 3. At the second dose level patients had to

**Figure 1** Study design.

receive C 1200 mg $m^{-2}$, D 55 mg $m^{-2}$, E 120 mg $m^{-2} \times 3$ and, at the third level, C 1300 mg $m^{-2}$, D 60 mg $m^{-2}$, E 130 mg $m^{-2} \times 3$. All patients received G-CSF (filgastrim) at the dose of 5 $\mu$g $kg^{-1}$ self-administered s.c. from day 5 to day 11. If GCPF or infection were found as the DLT, the protocol established that another cohort of patients at the same dose level would have been treated with the addition of anti-microbial prophylaxis from day 5 to day 11, assuming GCPF was caused by occult bacterial, fungal or viral infection (Figure 1). Anti-microbial prophylaxis had to consist of ciprofloxacin 500 mg twice a day, fluconazole 100 mg and acyclovir 800 mg twice a day (Table I).

Chemotherapy courses were repeated every 14 days if complete haematological recovery occurred (WBC > $3.0 \times 10^9$ $l^{-1}$ or granulocytes > $2.0 \times 10^9$ $l^{-1}$, platelets > $100 \times 10^9$ $l^{-1}$ and in the absence of any more than grade I non-haematological toxicity. In the case of an inadequate recovery from toxicity, patients were rechecked daily in order to resume treatment as soon as clinical and haematological conditions permitted. Chemotherapy had to be given for a minimum of three courses. No dose escalation was permitted until all the patients in the previous dose level had completed three courses of treatment.

No dose reduction was foreseen; if a DLT ocurred, the patient had to be removed from the study and to continue the treatment with a standard CDE (C 1000 mg $m^{-2}$ day 1, D 45 mg $m^{-2}$ day 1 and 100 mg $m^{-2}$ days 1, 2 and 3 every 21 days).

Patients who did not experience DLTs during the first three cycles were offered one or two more cycles of therapy. As supportive therapy, all patients with granulocytes < $0.5 \times 10^9$ $l^{-1}$ were given oral antibiotic prophylaxis with ciprofloxacin 500 mg twice a day and fluconazole 100 mg daily. In the case of GCPF, FUO or documented infection, an outpatient empirical antibiotic therapy consisting of ceftazidime 1 g three times a day intramuscularly was started. Packed RBCs were administered with Hb levels < 8 g $l^{-1}$ and platelets were transfused to maintain a platelet count > $15 \times 10^9$ $l^{-1}$.

### *Patient evaluation and follow-up*

During treatment complete blood cell counts and differentials were obtained every other day. In case a grade IV haematological toxicity was documented, blood counts were performed daily until recovery of grade IV toxicity. Clinical examination, recording of toxicity, performance status, weight and blood chemistry were performed before starting each chemotherapy cycle.

Patients were instructed to measure their axillary body temperature at least three times a day. In case of fever > 38.2°C they had a contact their physician as soon as possible. At the hospital, axillary temperature was taken again and a complete blood count obtained; if the temperature was > 38.2°C and the granulocytes were < 500 $ml^{-1}$ the patient was considered as having a GCPF. In this case routine cultures of blood, urine and other suspected sources were performed and empirical parenteral antibiotic treatment started. Evaluation of response was due after three cycles with CT scan of thorax and repetition of all previously positive tests. Patients with limited disease, achieving partial or complete response, received thoracic radiotherapy and only those in complete response were enrolled into a prospective multicentre randomised study of prophylactic cranial irradiation.

### *Response criteria and dose intensity calculation*

Standard WHO criteria for response and toxicity evaluation were used (Miller *et al.*, 1981). Dose intensity was expressed in mg $m^{-2}$ $day^{-1}$ and calculated according to Hryniuk and Bush (1984). As reference standard regimen to calculate the 'relative' dose intensity (RDI) planned at each dose level, a standard dose intensity CDE (C 1000 mg $m^{-2}$, D 45 mg $m^{-2}$, E $100 \times 3$ mg $m^{-2}$ every 21 days) was used. The reported dose intensity was the average (ARDI) of the three drugs used (C, D and E). The increase over the standard in the planned dose intensity at each dose level has been calculated as follows:

$$\frac{\text{Dose m}^{-2}\text{ at a given dose level} \times 3\text{ (No. of planned courses)}}{\text{No. of weeks elapsing between the first and the third course}} :$$

$$\frac{\text{Standard dose m}^{-2} \times 3}{\text{No. of weeks elapsing between the first and the third course}}$$

As an example, the increase in cyclophosphamide dose intensity at the first dose level is reported:

$$\frac{1100 \times 3}{4} : \frac{1000 \times 3}{6} = 825 : 500 = 1.65$$

The actual dose intensity was calculated at the third and at the fourth cycle. All patients were considered for actual dose intensity analysis including the patients who had to stop

**Table I** Dose escalation programme

| | *First level* | *Second level* | *Second level (b)* | *Third level* | *Third level (b)* |
|---|---|---|---|---|---|
| Cyclophosphamide[a] (mg $m^{-2}$ day 1) | 1100 | 1200 | 1200 | 1300 | 1300 |
| Doxorubicin[a] (mg $m^{-2}$ day 1) | 50 | 55 | 55 | 60 | 60 |
| Etoposide[a] (mg $m^{-2}$ days 1–3) | 110 | 120 | 120 | 130 | 130 |
| Filgastrim (5 $\mu$g $kg^{-1}$ days 5–11) | Yes | Yes | Yes | Yes | Yes |
| Ciprofloxacin (500 mg $\times$ 2 days 5–11) | No | No | Yes | Yes | Yes |
| Fluconazole (100 mg days 5–11) | No | No | No | No | Yes |
| Acyclovir (800 mg $\times$ 2 days 5–11) | No | No | No | No | Yes |
| Dose intensity (DI) increase[b] | 65% | 80% | 80% | 95% | 95% |

[a]Cycles of chemotherapy repeated every 14 days. [b]Increase in planned DI compared with a standard CDE (C 1000, D 45, E 100 $\times$ 3 every 21 days). See 'Patients and methods' for the calculation of dose-intensity increase.

treatment before the third or the fourth cycle. In these patients the total dose of chemotherapy received was divided by the time required to complete three or four cycles of therapy (4 and 6 weeks respectively), regardless of the number of courses actually received.

### Results

From February 1992 to June 1994 25 SCLC patients were enrolled into the study. Patient characteristics are shown in Table II. A total of 73 cycles, with a median of three (range 1–5) cycles per patient, were administered according to protocol.

### *Dose-limiting toxicity*

All the three patients enrolled at the first dose level completed three or more cycles of chemotherapy at the dose and schedule planned by the protocol and no DLT was seen.

**Table II** Patient characteristics and response to treatment

| | |
|---|---|
| No. of patients | 25 |
| Median age (range) | 58 (41–69) |
| Sex (M/F) | 21/4 |
| Median PS (range) | 0 (0–1) |
| Stage | |
| LD | 12 |
| ED | 13 |
| Response | |
| CR | 8 |
| PR | 13 |
| NE | 4[a] |

[a]One patient treated as adjuvant therapy after surgery, one patient lost to follow-up before evaluation and two patients died before evaluation.

At the second dose level, three out of five patients had a DLT. Two had GCPF after the first or second cycle and one had GCPF with a clinically documented infection (dental abscess) after the second cycle. However, the duration of GCPF was only 1 day in all the patients and no systemic infection was documented.

According to the protocol, the enrolment of patients was resumed at the same dose level adding prophylactic antibiotics consisting of ciprofloxacin 500 mg twice a day from day 5 to 11. Six more patients were treated at this dose level and only two had a DLT. One patient had a microbiologically documented infection (oral candidiasis) after the second cycle and one patient had one episode of FUO lasting for 6 days associated with stomatitis and balanoproctitis after the third cycle. The second dose level, with the addition of prophylactic ciprofloxacin, was therefore considered feasible and patient accrual proceeded to the third dose level using ciprofloxacin in adjunct to G-CSF prophylaxis. Four out of six patients experienced a DLT. One patient had GCPF and a microbiologically documented infection (oral candidiasis) after the first cycle; another had a thoracic herpes zoster infection without GCPF after three cycles; the third developed GCPF before the second cycle; and the last patient had GCPF with generalised mucositis (stomatitis and balanoproctitis) during the second cycle. Based on the observation that, with the addition of ciprofloxacin prophylaxis, all documented infections seen in our study were non-bacterial, another cohort of patients was treated at the third dose level adding to ciprofloxacin anti-fungal (fluconazole 100 mg daily) and anti-viral (acyclovir 800 mg twice a day) prophylaxis from day 5 to 11. Five more patients were accrued of which three experienced a DLT. One patient had GCPF and a clinically documented infection (pneumonia) after the second cycle; he died 20 days later with leucocytosis (WBC = $56.0 \times 10^9\ l^{-1}$) and multiple cerebral ischaemic lesions revealed at brain CT scan. Another patient had grade IV leucopenia lasting for 5 days along with grade IV thrombocytopenia requiring platelet transfusion and hospital admission for 6 days. The third patient with DLT had GCPF without documented infection during the third course (Table III).

**Table III** Results

| | *Patients* | *No. of courses* | *GCPF* | *No. of days with GCPF or FUO* | *Infection* | *DLT* |
|---|---|---|---|---|---|---|
| | 1 | 5 | – | 0 | – | – |
| First level | 2 | 3 | – | 0 | – | – |
| | 3 | 4 | – | 0 | – | – |
| | 4 | 4 | – | 0 | – | – |
| | 5 | 3 | – | 0 | – | – |
| Second level | 6 | 2 | Yes | 1 | – | GCPF |
| | 7 | 1 | Yes | 1 | – | GCPF |
| | 8 | 2 | Yes | 1 | Dental abscess | GCPF/INF |
| | 9 | 4 | – | 0 | – | – |
| | 10 | 4 | – | 0 | – | – |
| | 11 | 3 | – | 0 | – | – |
| Second level (b) | 12 | 3 | Yes | 1 | Oral candidiasis | GCPF/INF |
| | 13 | 3 | – | 0 | – | – |
| | 14 | 3 | – | 6 | FUO/mucositis[a] | FUO |
| | 15 | 2 | Yes | 3 | Oral candidiasis | GCPF/INF |
| | 16 | 3 | – | 0 | – | – |
| | 17 | 4 | – | 0 | – | – |
| Third level | 18 | 4 | – | 0 | Zoster | INF |
| | 19 | 1 | Yes | 1 | – | GCPF |
| | 20 | 2 | Yes | 2 | Mucositis[a] | GCPF |
| | 21 | 3 | – | 0 | – | – |
| | 22 | 2 | Yes | 2 | Pulmonary | GCPF/NF |
| | 23 | 1 | – | 0 | – | WBC IV |
| Third level | 24 | 3 | Yes | 2 | – | GCPF |
| | 25 | 4 | – | 0 | – | – |

FUO, fever of unknown origin; WBC IV, grade IV leucopenia for 5 days; INF, infection; GCPF, granulocytopenic fever. [a]Stomatitis and balanoproctitis.

*Other toxicities*

The nadir haematological toxicity is shown in Table IV. At the first dose level no patient developed grade IV toxicity. At the second and third dose levels 22.5% and 31% of cycles produced grade IV leucopenia whose median duration was 2 (range 1–4) and 3 (range 1–5) days respectively. The median WBC, Hb and platelet nadirs tended to worsen with dose escalation but remained relatively constant throughout the first three cycles. Only one patient required platelet transfusion after the first cycle and no RBC transfusion had to be given during the first three cycles. Four patients out of eight who continued chemotherapy beyond the third cycle required RBC transfusions for the occurrence of ⩾grade 3 anaemia. Non-haematological toxicity was mild and in no instance did it exceed grade II with the exception of the two cases of generalised mucositis mentioned above. Particularly, no case of significant cardiac, renal or hepatic toxicity was reported.

*Dose intensity*

At all dose levels the median interval between cycles was 14 days (range 14–21 days). The percentages of cycles delivered on day 15, as scheduled by the protocol, at the three dose levels were 100%, 58% and 56% respectively. No dose reduction was applied. Seventeen patients were able to complete the planned three courses. Among these, seven and one patients, respectively, were given one and two more courses (Table III).

The median percentages of actually delivered *vs* planned dose intensity, calculated for all patients at the third cycle, were 96%, 85%, 82% in the three dose levels respectively. At the fourth cycle, they were 85%, 75% and 71% respectively.

*Response and survival*

Results in terms of objective response to treatment are reported in Table II. Four patients were not evaluable for response. One patient received chemotherapy as adjuvant treatment after surgery. One patient was lost to follow-up and two others died before response evaluation. Out of 21 evaluable patients there were eight complete responses and 13 partial responses. Median time to progression and median overall survival, calculated according to the method of Kaplan and Meier, were 44 and 68 weeks respectively.

### Discussion

Since the demonstration of the important role played by dose intensity in the treatment outcome of a number of drug-sensitive tumours (Gurney *et al.*, 1993) and the availability for clinical use of haemopoietic growth factors, phase I–II studies of chemotherapy intensification have abounded (Bronchud, 1993). The primary objective of these studies was to assess whether haemopoietic growth factors, by ameliorating chemotherapy haematological toxicity, were able to allow chemotherapy dose intensification. Increase of dose intensity above that of standard chemotherapy can be accomplished with both dose escalation and schedule acceleration. Since the main effect of GM- and G-CSF is in accelerating neutrophil recovery, and only to a lesser extent in reducing the severity of neutrophil nadir, we first aimed at verifying the possibility of accelerating chemotherapy administration. In a study of 15 SCLC patients we were able to deliver a CDE regimen, at standard doses, combined with GM-CSF every 2 weeks as opposed to the usual 3 weeks, resulting in a 50% projected dose intensity escalation (Ardizzoni *et al.*, 1993). Two subsequent randomised trials confirmed that such an acceleration was not possible in the absence of the use of GM-CSF (Ardizzoni *et al.*, 1994; Pennella *et al.*, 1995). The DLT of accelerated chemotherapy is cumulative thrombocytopenia which becomes severe, requiring platelet transfusions, after the fourth cycle and often precludes the completion of treatment.

In the present study we aimed at identifying the maximum tolerated dose intensity by exploring the feasibility of dose escalating a CDE regimen repeated at 2 week intervals on an outpatient basis for at least three courses. The decision to administer a fourth course of therapy was left open and was indeed taken, with no serious adverse events, in the majority of patients who did not develop a DLT. This number of courses seems adequate in the treatment of SCLC based on the results of a recent randomised British study (Bleehen *et al.*, 1993).

Given the definition of DLT used in our study, the MTD turned out to be the first dose level (C 1100 mg $m^{-2}$, D 50 mg $m^{-2}$, E 110 mg $m^{-2} \times 3$) which corresponds to a 65% projected dose intensity increase compared with a standard every 21 days CDE. This poor level of dose escalation, even in the presence of G-CSF support, is not surprising since a number of other phase I studies, most of which used GM-CSF as a haemopoietic growth factor, came to a similar conclusion in SCLC (Paccagnella *et al.*, 1993), breast (Hoekman *et al.*, 1991), ovarian (Rusthoven *et al.*, 1991) and urothelial cancer (Scher *et al.*, 1992). The lack of a significant impact of myelocytic growth factors on the depth of neutrophil nadir may account for this result.

At the second dose level three of five patients developed GCPF. However, GCPF lasted only 1 day in all patients and in no instance did it require parenteral antibiotic treatment or hospitalisation. Therefore, using less strict criteria for defining a DLT, the second dose level, corresponding to an 80% projected dose intensity increase, might also be considered feasible. This amount of dose intensity increase is probably the maximum achievable on an outpatient basis, with a strategy of combined dose escalation and schedule acceleration using haemopoietic growth factors as the only supportive treatment. Further dose intensification is hindered by the development of GCPF and infection.

The efficacy of antibiotic prophylaxis in preventing infections in patients undergoing conventional chemotherapy for solid tumours is still '*sub judice*'. Previous trials of prophylactic cotrimoxazole as adjunct to chemotherapy of SCLC have shown moderate benefit in reducing the infection rate (Figueredo *et al.*, 1985; de Jongh *et al.*, 1983). Quinolones have been found to be more effective than cotrimoxazole in the prevention of infectious complications in the treatment of haematological malignancies (Dekker *et al.*, 1987). As yet no such study is available for solid tumours.

In our study, the addition of ciprofloxacin at the second

**Table IV** Nadir haematological toxicity

| | *Media WBC* $\times 10^9$ $l^{-1}$ *(range)* | | | *Media Hb g* $dl^{-1}$ *(range)* | | | *Median platelets* $\times 10^9$ $l^{-1}$ *(range)* | | |
|---|---|---|---|---|---|---|---|---|---|
| *Cycles* | *1* | *2* | *3* | *1* | *2* | *3* | *1* | *2* | *3* |
| First level | 3.0<br>(1.4–3.4) | 2.0<br>(1.2–2.9) | 2.1<br>(2.0–2.5) | 12.8<br>(12.6–13.5) | 12.3<br>(11.5–12.5) | 12.1<br>(9.5–12.5) | 140<br>(115–180) | 120<br>(100–148) | 100<br>(98–150) |
| Second level | 1.5<br>(0.5–2.9) | 0.9<br>(0.3–1.8) | 1.8<br>(0.8–2.8) | 11.5<br>(8.6–13.8) | 10.1<br>(8.2–13.5) | 9.3<br>(7.5–13.5) | 100<br>(30–280) | 88<br>(56–173) | 85<br>(65–140) |
| Third level | 1.0<br>(0.4–2.1) | 1.2<br>(0.3–1.5) | 1.8<br>(0.5–2.1) | 12.1<br>(9.1–14.5) | 10.8<br>(7.8–12.4) | 10.3<br>(8.2–11.1) | 98<br>(9–184) | 101<br>(40–203) | 118<br>(80–202) |

dose level almost abrogated the occurrence of GCPF. The small number of patients does not allow us to draw firm conclusions on the role of quinolone prophylaxis in this setting. However, since GCPF remains the dose-limiting toxicity of dose-intensified CDE, despite the use of G-CSF, the efficacy of antibiotic prophylaxis in conjunction with haemopoietic growth factors deserves further exploration with specific trials. Given the increasing prevalence of Gram-positive infection in patients receiving prophylactic quinolones (Meunier, 1990), ciprofloxacin alone may not be the optimal antibiotic treatment and the combination of ciprofloxacin with other agents such as vancomycin, rifampicin or roxithromycin is currently advocated (Archimbaud *et al.*, 1991). Most patients receiving G-CSF plus ciprofloxacin at the second and third dose level developed non-bacterial infections or mucositis. Both fluconazole and acyclovir have been found to be effective in preventing, respectively, fungal and viral infections in patients undergoing intensive anti-neoplastic therapy for the treatment of haematological malignancies (Wade *et al.*, 1984; Winston *et al.*, 1993). Despite the use of anti-mitotic and anti-viral prophylactic therapy, the third dose level did not appear feasible in our study owing again to the occurrence of severe leucopenia and fever. However, none of the patients developed mucositis or oral candidiasis, which, on the contrary, was frequently observed in previous patient cohorts where no anti-fungal or anti-viral prophylaxis was used. Also this observation, owing to the small sample size and to the type of study design, only permits one to hypothesise on the possible role of fluconazole and acyclovir prophylaxis in moderately dose-intensive treatments for solid tumours which would need to be addressed with more appropriate studies.

In conclusion, with a simultaneous dose escalation and schedule acceleration it is indeed possible to take maximum advantage of G-CSF activity and to increase CDE dose intensity by a factor 1.65–1.80 for a maximum of 3–4 courses. The next step is to assess the impact of such a dose intensity increase on the treatment outcome of SCLC patients. A randomised prospective study comparing standard CDE *vs* CDE at the MTD identified in the present study is presently ongoing in Europe. The study is also designed to address the role of antibiotic prophylaxis in this setting (Tjan-Heijnen *et al*).

**Acknowledgements**
We are indebted to Marina Di Dino for reviewing the English of the manuscript and to Giuliana Vaglio for her help in the preparation of tables and figures. We also thank Amgen-Roche Italy for the supply of G-CSF.

### References

ARCHIMBAUD E, GUYOTAT D, MAUPAS J, PLOTON C, NAGEOTTE A, DEVAUX V, THOMAS X, FLEURETTE J AND FIERE D. (1991). Pefloxacin and vancomycin versus gentamicin, colistin sulphate and vancomicin for prevention of infections in granulocytopenic patients: a randomised double-blind study. *Eur. J. Cancer*, **27**, 174–178.

ARDIZZONI A, VENTURINI M, CRINO' L, SERTOLI MR, BRUZZI P, PENNUCCI MC, MARIANI GL, GARRONE O, BRACARDA S, ROSSO R AND VAN ZANDWIJK N. (1993). High dose-intensity chemotherapy, with accelerated cyclophosphamide–doxorubicin–etoposide and granulocyte–macrophage colony stimulating factor, in the treatment of small cell lung cancer. *Eur. J. Cancer*, **29A**, 687–692.

ARDIZZONI A, VENTURINI M, SERTOLI MR, GIANNESSI PG, BREMA F, DANOVA M, TESTORE F, MARIANI GL, PENNUCCI MC, QUEIROLO P, SILVESTRO S, BRUZZI P, LIONETTO R, LATINI F AND ROSSO R. (1994). Granulocyte-macrophage colony stimulating factor (GM-CSF) allows acceleration and dose-intensity of CEF chemotherapy: a randomised study in patients with advanced breast cancer. *Br. J. Cancer*, **69**, 385–391.

ARRIAGADA R, LECHEVALIER T, PIGNON JP, RIVIERE A, MONNET I, CHOMY P, TUCHAIS C, TARAYRE M AND RUFFIE P. (1993). Initial chemotherapeutic doses and survival in patients with limited small-cell lung cancer. *N. Engl. J. Med.*, **329**, 1848–1852.

BLEEHEN NM, GIRLING DJ, MACHIN D, STEPHENS RJ, BOLGER JJ, CLARK PI AND HASLETON PS. (1993). A randomised trial of three or six courses of etoposide cyclophosphamide methotrexate and vincristine or six courses of etoposide and ifosfamide in small cell lung cancer (SCLC) I: survival and prognostic factors. *Br. J. Cancer*, **68**, 1150–1156.

BRONCHUD M. (1993). Can hematopoietic growth factors be used to improve the success of cytotoxic chemotherapy? *Anti-Cancer Drugs*, **4**, 127–139.

CRAWFORD J, OZER H, STOLLER R, JOHNSON D, LYMAN G, TABBARA I, KRISS M, GROUS J, PICOZZI V, RAUSCH G, SMITH R, GRADISHAR W, YAHANDA A, VINCENT M, STEWART M AND GLASPY J. (1993). Reduction by granulocyte colony-stimulating factor of fever and neutropenia induced by chemotherapy in patients with small cell lung cancer. *N. Engl. J. Med.*, **325**, 164–170.

DE JONGH CA, WADE GC, FINLEY RS, JOSHI JH, AISNER J, WIERNIK PH AND SCHIMPFF C. (1983). Trimethoprim/sulfamethoxazole versus placebo: a double-blind comparison of infection prophylaxis in patients with small cell carcinoma of the lung. *J. Clin. Oncol.*, **1**, 302–307.

DEKKER AW, ROZEMBERG-ARSKA MA AND VERHOEF J. (1987). Infection prophylaxis in acute leukemia: a comparison of ciprofloxacin with trimethoprim-sulfamethoxazole and colistin. *Ann. Int. Med.*, **106**, 7–12.

FIGUEREDO AT, HRYNIUK WA, STRAUTMANIS I, FRANK G AND RENDELL S. (1985). Co-trimoxazole prophylaxis during high-dose chemotherapy of small-cell lung cancer. *J. Clin. Oncol.*, **3**, 54–64.

GURNEY H, DODWELL D, THATCHER N AND TATTERSALL MHN. (1993). Escalating drug delivery in cancer therapy: a review of concepts and practice. II. *Ann. Oncol.*, **4**, 103–115.

HAMM J, SCHILLER JH, CUFFIE C, OKEN M, FISHER RI, SHEPHERD F AND KAISER G. (1994). Dose-ranging study of recombinant human granulocyte-macrophage colony-stimulating factor in small cell lung carcinoma. *J. Clin. Oncol.*, **12**, 2667–2676.

HOEKMAN K, WAGSTAFF J, VAN GROENINGEN CJ, VERMORKEN JB, BOVEN E AND PINEDO HM. (1991). Effect of recombinant human granulocyte-macrophage colony-stimulating factor on myelosuppression induced by multiple cycles of high-dose chemotherapy in patients with advanced breast cancer. *J. Natl Cancer Inst.*, **3**, 1546–1553.

HRYNIUK W AND BUSH H. (1984). The importance of dose intensity in chemotherapy of metastatic breast cancer. *J. Clin. Oncol.*, **2**, 1281–1288.

IHDE DC. (1992). Drug therapy: chemotherapy of lung cancer. *N. Engl. J. Med.*, **327**, 1434–1441.

KLASA RJ, MURRAY N AND COLDMAN AJ. (1991). Dose-intensity meta-analysis of chemotherapy regimens in small-cell carcinoma of the lung. *J. Clin. Oncol.*, **9**, 499–508.

MEUNIER F. (1990). Prevention of infection in neutropenic patients with pefloxacin. *J. Antimicrob. Chemother.*, **26**, 69–73.

MILLER AB, HOOGSTRATEN AB, STRAQUET M AND WINKLER A. (1981). Reporting results of cancer treatment. *Cancer*, **47**, 207–214.

OHRLING M, BJORKHOLM M, OSTERBORG A, JULISSON G, BJOREMAN M, CARLSON K, CELSING F, GAHRTON G, GRIMFORS G, GRUBER A, GYLLENHAMMAR H, HAST R, JOHANSSON B, JARNMARK M, KIMBY E, LERNER R, LINDER O AND MELLSTEDT H. (1993). Etoposide, doxorubicin, cyclophosphamide and high-dose betamethasone (EACB) as an outpatient salvage therapy for refractory multiple myeloma. *Eur. J. Haematol.*, **51**, 45–49.

PACCAGNELLA A, FAVARETTO A, RICCARDI A, DANOVA M, GHIOTTO C, GIORDANO M, PAPPAGALLO G, COMIS S, PANOZZO M, CHIECO-BIANCHI L AND FIORENTINO MV. (1993). Granulocyte-macrophage colony stimulating factor increases dose-intensity of chemotherapy in small cell lung cancer. *Cancer*, **72,** 697–706.

PENNELLA JW, HODGETTS J, LOMAX L, BILDET F, COURCHABERNAUD V AND THATCHER N. (1995). Can cytotoxic dose-intensity be increased by using granulocyte colony-stimulating factor? A randomized controlled trial of lenograstim in small-cell lung cancer. *J. Clin. Oncol.*, **13,** 652–659.

RUSTHOVEN J, LEVIN L, EISENHAUER E, MAZURKA J, CARMICHAEL J, O'CONNEL G, BRYSON P, HIRTE H AND KOSKI B. (1991). Two phase I studies of carboplatin dose-escalation in chemotherapy-naive ovarian cancer patients supported with granulocyte-macrophage colony-stimulating factor. *J. Natl Cancer Inst.*, **83,** 1748–1753.

SCHERR HI, SIEDMAN AD, BAJORIN DF, MOTZER RJ, CURLEY T, O'DELL M, QUINLIVAN S, FAIR W, HERR H, SHEINFELD J, SOGANI P, RUSSO P, DERSHAW DD, TAO Y, BEGG C AND BOSL GJ. (1992). Escalated methotrexate, vinblastine, adriamycin and cisplatin (E-MVAC) with granulocyte colony stimulating factor (G-CSF) in urothelial cancer (abstract 610). *Proc. Am. Soc. Clin. Oncol.*, **11,** 199.

SOMLO G, DOROSHOW JH, FORMAN SJ, LEONG LA, MARGOLIN KA, MORGAN RJ, RASCHKO JW, AKMAN SA, AHN C, NAGASAWA S AND HARRISON J. (1994). High dose doxorubicin, etoposide and cyclophosphamide with stem cell reinfusion in patients with metastatic or high-risk primary breast cancer. *Cancer*, **73,** 1678–1685.

SPARANO JA, WIERNIK PH, LEAF A AND DUTHER JP. (1993). Infusional cyclophosphamide, doxorubicin, and etoposide in relapsed and resistant non-Hodgkin's lymphoma: evidence for a schedule-dependent effect favoring infusional administration of chemotherapy. *J. Clin. Oncol.*, **11,** 1071–1079.

STEWARD WP, VON PAWEL J, GATZEMEIER U, THATCHER N AND FRISH J FOR SCLC STUDY GROUP. (1995). Dose-intensification of V-ICE chemotherapy with GM-CSF in small cell lung cancer (SCLC). A prospective randomised study of 301 patients. *Eur. J. Cancer*, (Suppl. 5), **31A,** S18.

TJAN-HEIJNEN VCG, POSTMUS PE AND ARDIZZONI A. (1994). A phase III study of standard CDE versus intensified CDE with G-CSF (filgrastim) with or without prophylactic antibiotics in small cell lung cancer (SCLC). EORTC-LCCG Clinical Study Protocol 08923.

TRILLET-LENOIR V, GREEN J, MANEGOLD C, VON PAWEL J, GATZEMEIER U, LEBEAU B, DEPIERRE A, JOHNSON P, DECOSTER G, TOMITA D AND EWEN C. (1993). Recombinant granulocyte colony stimulating factor reduces the infectious complications of cytotoxic chemotherapy. *Eur. J. Cancer*, **29,** 319–324.

WADE JC, NEWTON B, FLOURNOY N AND MEYERS JD. (1984). Oral acyclovir for the prevention of herpes symplex virus reactivation after marrow transplatation. *Ann. Int. Med.*, **100,** 823–828.

WINSTON DJ, CHANDRASEKAR PH, LAZARUS HM, GOODMAN JL, SILBER JL, HOROWITZ H, SHADDUCK RK, ROSENFELD CS, HO WG, ISLAM MZ AND BUELL DN. (1993). Fluconazole prophylaxis of fungal infections in patients with acute leukemia. Results of a randomized placebo-controlled, double-blind, multicenter trial. *Ann. Int. Med.*, **118,** 495–503.